# mSphere of Influence: The power of observational research

**DOI:** 10.1128/msphere.00176-24

**Published:** 2024-07-02

**Authors:** Katrina B. Velle

**Affiliations:** 1 Department of Biology, University of Massachusetts Dartmouth, North Dartmouth, Massachusetts, USA; University at Buffalo, Buffalo, New York, USA

**Keywords:** microscopy, actin, *Listeria*

## Abstract

Katrina Velle is a cell biologist who uses microscopy to study amoebae. In this mSphere of Influence article, she reflects on how a classic paper on *Listeria* by Tilney and Portnoy made an impact on her by highlighting how much we can learn from simply looking at cells.

## COMMENTARY

There is power in looking at cells on a microscope. This is how the fields of cell biology and microbiology began—with scientists like Hooke and van Leeuwenhoek discovering “cells” (1) and “wee beasties” (2) on early microscopes and describing them in such exquisite detail that we understand today what they saw 350 years ago (3). In these early days of microscopy, without cameras and the ability to pause and rewind, scientists had to be experts at observing, describing, and drawing. There is now less value placed on simply observing samples; we can take thousands of images a day, automate analysis workflows, and write entire papers without spending more than a few seconds looking at any individual cell. But, observation remains an incredibly powerful tool and is still leading to new, unexpected discoveries [e.g., (4–6)]. Observation is particularly critical for understudied organisms and emerging model systems, where we sometimes do not know what we are looking for. A stunning example of this is Tilney and Portnoy’s classic JCB paper, “Actin Filaments and the Growth, Movement, and Spread of the Intracellular Bacterial Parasite, *Listeria monocytogenes*” (7).

While *Listeria* is now the textbook model for microbes that hijack host cell machinery, almost nothing was known about how these intracellular bacteria interact with host cells when Tilney and Portnoy took to their microscope. The experiment was straightforward; they infected mammalian macrophages with *Listeria* for short or long incubations, then imaged the infected cells using transmission electron microscopy. What they saw was amazing. Some bacteria had clouds of actin meshworks surrounding them, while others had long actin networks forming “comet tails” at one pole. Some of these comet tails extended far from the surface of the cell, and others were encapsulated by layers of host membrane. By carefully organizing these images, Tilney and Portnoy were able to take fixed points in infection and propose a model for how *Listeria* spread from cell to cell; bacteria escape their initial vacuole, form an actin comet tail, protrude into an adjacent cell until they are engulfed, then break down the layers of host cell membrane to start the process again. The authors put this finding best, “Thus, this insidious beast has managed to multiply and spread cell-to-cell without leaving the cytoplasm of its host.” Three decades later, the model they created in this publication (Figure 23) is still shown in *Listeria* talks.

Reading this article as a graduate student taught me an important lesson: you do not know what you are missing if you do not look. No one would have guessed that *Listeria* build actin comet tails to rocket around the cytosol and spread from cell to cell before this paper, but in the following years, it became a foundational model for the study of actin networks (8, 9). This has inspired my own work, as I intentionally take time to look at the cells I image on the microscope and frequently return to old images for a second look. If not for careful observation, I would not have noticed how pathogenic *E. coli* were spreading from cell to cell for my first paper as a graduate student, nor would I have defined how *Naegleria* amoebae crawl as a postdoc. Now, as I begin training students in my own lab, I encourage them to spend time looking at cells to hone their observational skills. The importance of looking at cells cannot be understated; after all, van Leeuwenhoek could not have imagined the diverse world of bacteria and protists living in water samples until he thought to look.
